# Supplementary material for: Enhancing childhood immunization coverage in Mozambique and Malawi: Study protocol of a mixed methods evaluation of the ‘Let’s talk about vaccines’ multisite community-based participatory project
Source: PLoS One. 2024 Nov 20;19(11):e0311052. doi: 10.1371/journal.pone.0311052 (PMC11578484; doi:10.1371/journal.pone.0311052)
Supplement: Attachment — Submitted filename: PLOS ONE_Linda Shuro et al., 2024_Lets talk about vaccines.docx [file pone.0311052.s002.docx]

**Enhancing Childhood Immunization Coverage in Mozambique and Malawi: Study**

**Protocol of a mixed methods evaluation of the Let's Talk About Vaccines Multisite**

**Community-Based Participatory Project**

General comments

Vaccinations remain a major advancement in global public health, despite challenges for these live-saving vaccines to reach a majority of children at need in hard-to-reach zones. It is important to understand community-level drivers on vaccine uptake needed to support health decision making about prioritization and cost of these interventions in one’s own setting.

This study protocol aims to evaluate the effectiveness (on under-two immunization coverage) and the extent of implementation (reach, adoption, implementation and maintenance) of the Project in Mozambique and Malawi.

Authors plan to integrate the theory of change approach with the RE-AIM framework, and hope findings will potentially provide evidence on mechanisms that explain observed improvements in under two immunization outcomes and toward resilient health systems as a result of the co-created community driven solution(s).

The paper will bring important addition to existing literature, especially from an area where data paucity remains a perennial issue. However, this reviewer finds that there are gaps to address in the current version of the paper before it is considered for publication.

SPECIFIC COMMENTS

Title: No need to capitalize each word, only names of places and persons here except that falls within the journal’s specifications.

-	The manuscript lacks line numbering for easy reading and review. Also, there is need for authors to format the entire work appropriately.

ABSTRACT

- CBPR: Please after first use of an abbreviation, it should be used thereafter. No need spelling out in full.
- “There is a few example of evaluations of intervention created through human-centred design.” Authors can integrate this in the next sentence i.e., “… this approach...” or take it to the introduction.

INTRODUCTION

- “It is an approach that centres on creativity in an interactive process to bring human centred views … “Here, I think it gives a wrong impression that previous interventions have not been human-centred. This should be revised to community-directed/driven/centred.
- “… by evaluating the effectiveness and implementation of the project.” How will these be performed?
- Ensure consistency in reporting (e.g., RE-AIM or RE AIM)
- In figures/ Tables, authors should ensure that each abbreviations should be fully defined as footnotes if not within the figure (s)/Table(s) e.g., what is TOC, VR, HCD?
- DPT1-Penta and all abbreviations should be fully defined at first use before subsequent uses.
- Could the authors describe how data quality will be assessed considering the porous nature of data in resource-low settings?
- No need to put “long-term impacts” in bold!
- Figure 2 is very illustrative and it’s great! Except that some of the arrows are out of place or non-directional. Please, verify and adjust!
- What’s the rationale of targeting under-2 populations? Would this be sufficient to assess the long-term impacts?
- Ethics statement should only appear in the text (not abstract, except the journal demands that).

DISCUSSION/Limitation

- Incomplete records are a perennial problem in resource-low setting, how do the authors plan to ascertain data quality with this huge challenge?
- Additionally, authors need to explain clearly how they plan to overcome the stated limitation so as not to jeopardize potential findings.

REFERENCES

- Some are wrongly captured and should be verified at the level of the reference manager before uploading, so that articles should be referenced appropriately.
