# Supplementary material for: Enhancing childhood immunization coverage in Mozambique and Malawi: Study protocol of a mixed methods evaluation of the ‘Let’s talk about vaccines’ multisite community-based participatory project
Source: PLoS One. 2024 Nov 20;19(11):e0311052. doi: 10.1371/journal.pone.0311052 (PMC11578484; doi:10.1371/journal.pone.0311052)
Supplement: Attachment — Submitted filename: Response to Reviewers- PLOS ONE.docx [file pone.0311052.s003.docx]

26 July 2024

Dear PLOS ONE Editor

**RE: Response to Reviewers- PLOS ONE**

Thank you for the opportunity to revise and resubmit our manuscript titled **“Enhancing childhood immunization coverage in Mozambique and Malawi: Study Protocol of a mixed methods evaluation of the ‘Let's Talk About Vaccines’ multisite community-based participatory project**”.

Thank you for reviewing our proposal and providing us with further valuable feedback to strengthen our manuscript. We have carefully considered the editor’s and reviewers’ comments and have made substantial changes to address their concerns. Below is a detailed, point-by-point response for each of the suggested aspects and comments. The reviewers’ comments are in bold and our responses are in normal font.

**Editor’s comments**

1. **Please ensure that your manuscript meets PLOS ONE's style requirements, including those for file naming.**

Detailed revisions have been made accordingly.

2. **Please provide a complete Data Availability Statement in the submission form, ensuring you include all necessary access information or a reason for why you are unable to make your data freely accessible. If your research concerns only data provided within your submission, please write "All data are in the manuscript and/or supporting information files" as your Data Availability Statement.**

The statements " No datasets were generated in this study protocol. All relevant data from this study will be made available upon study completion." has been added to the Data Availability Statement**.** See pg. 26

3. **Your ethics statement should only appear in the Methods section of your manuscript. If your ethics statement is written in any section besides the Methods, please move it to the Methods section and delete it from any other section. Please ensure that your ethics statement is included in your manuscript, as the ethics statement entered into the online submission form will not be published alongside your manuscript.**

The ethics statement has been moved to the Methods section. See pg. 18

4. **Please include captions for your Supporting Information files at the end of your manuscript, and update any in-text citations to match accordingly. Please see our Supporting Information guidelines for more information:** [**http://journals.plos.org/plosone/s/supporting-information**](http://journals.plos.org/plosone/s/supporting-information) **.**

The captions for the Supporting information files have been included at the end of the manuscript and in-text citations matched accordingly.

5. **Please review your reference list to ensure that it is complete and correct. If you have cited papers that have been retracted, please include the rationale for doing so in the manuscript text, or remove these references and replace them with relevant current references. Any changes to the reference list should be mentioned in the rebuttal letter that accompanies your revised manuscript. If you need to cite a retracted article, indicate the article’s retracted status in the References list and also include a citation and full reference for the retraction notice.**

We have revised the reference list to ensure that it is complete and correct using the Mendeley reference manager.

Reviewer #1: General comments

**Vaccinations remain a major advancement in global public health, despite challenges for these live-saving vaccines to reach a majority of children at need in hard-to-reach zones. It is important to understand community-level drivers on vaccine uptake needed to support health decision making about prioritization and cost of these interventions in one’s own setting.**

**This study protocol aims to evaluate the effectiveness (on under-two immunization coverage) and the extent of implementation (reach, adoption, implementation and maintenance) of the Project in Mozambique and Malawi.**

**Authors plan to integrate the theory of change approach with the RE-AIM framework, and hope findings will potentially provide evidence on mechanisms that explain observed improvements in under two immunization outcomes and toward resilient health systems as a result of the co-created community driven solution(s).**

**The paper will bring important addition to existing literature, especially from an area where data paucity remains a perennial issue. However, this reviewer finds that there are gaps to address in the current version of the paper before it is considered for publication.**

Thank you for the positive feedback and insightful comments on our study protocol.

SPECIFIC COMMENTS

**Title: No need to capitalize each word, only names of places and persons here except that falls within the journal’s specifications.**

We have revised the title to ensure only proper nouns are capitalized.

**The manuscript lacks line numbering for easy reading and review. Also, there is need for authors to format the entire work appropriately**.

We have added page numbers to facilitate easier reading and review. Additionally, we have reformatted the entire manuscript.

ABSTRACT

**CBPR: Please after first use of an abbreviation, it should be used thereafter. No need spelling out in full**.

Duly noted and revised. However, we have repeated spelling out in full in the main article from the introduction.

**“There is a few example of evaluations of intervention created through human-centred design.” Authors can integrate this in the next sentence i.e., “… this approach...” or take it to the introduction.**

We have deleted the sentence as we already have a similar sentence in the introduction.

INTRODUCTION

**“It is an approach that centres on creativity in an interactive process to bring human centred views … “Here, I think it gives a wrong impression that previous interventions have not been human-centred. This should be revised to community-directed/driven/centred.**

Duly noted and revised to community-driven. See pg. 3

**“… by evaluating the effectiveness and implementation of the project.” How will these be performed?**

We have revised to include how this will be performed leading to the next section on the methodology. See pg. 5

**Ensure consistency in reporting (e.g., RE-AIM or RE AIM)**

Duly noted and revised in all sections.

**In figures/ Tables, authors should ensure that each abbreviations should be fully defined as footnotes if not within the figure (s)/Table(s) e.g., what is TOC, VR, HCD?**

Noted and revised accordingly.

**DPT1-Penta and all abbreviations should be fully defined at first use before subsequent uses.**

All abbreviations have been fully defined at first use.

**Could the authors describe how data quality will be assessed considering the porous nature of data in resource-low settings?**

We acknowledge the challenges associated with data quality in resource-low settings and have included a detailed description of our plan to assess and ensure data quality throughout our study. See pg.15

**No need to put “long-term impacts” in bold!**

Noted and revised on pg. 22

**Figure 2 is very illustrative and it’s great! Except that some of the arrows are out of place or non-directional. Please, verify and adjust!**

The arrows intertwined were placed to show the interconnectedness of the process as it unfolds and any feedback loops. However, we have revised the figure so that it can be easily understood. See Fig 1. We have revised the figure to be figure 1 to follow a chronological order.

**What’s the rationale of targeting under-2 populations? Would this be sufficient to assess the long-term impacts?**

This is the standard timeframe looked at for assessing routine immunization coverage and key indicators as most children should receive all required vaccination by the age of 2. This is also aligned with the recommended vaccination schedule for both Mozambique & Malawi <https://dhsprogram.com/pubs/pdf/CS1/CS1.pdf>. We have included similar statements on pg. 15

**Ethics statement should only appear in the text (not abstract, except the journal demands that).**

Noted and removed from the abstract and moved to the methods section. See pg. 18

DISCUSSION/Limitation

**Incomplete records are a perennial problem in resource-low setting, how do the authors plan to ascertain data quality with this huge challenge?**

We have included a detailed description of our plan to assess and ensure data quality throughout our study and document any anomalies considering this perennial problem. See pg.15. We have also included a similar statement under limitations which also explains how we will overcome the stated limitations. See pg. 25

**Additionally, authors need to explain clearly how they plan to overcome the stated limitation so as not to jeopardize potential findings.**

We have also explained this under data collection and limitations.

REFERENCES

**Some are wrongly captured and should be verified at the level of the reference manager before uploading, so that articles should be referenced appropriately**

References revised using Mendeley reference manager.

**Reviewer #2 comments:**

**The standardization of the caretaker researchers and the control sites is missing.**

Standardization of caregiver researchers is explained on page 9 in the inclusion criteria. However, caregiver researchers are participants in this evaluation and not researchers for this evaluation. A description of the selection criteria of the control sites has been added. The selection of control sites will be conducted after the CBPR and HCD approaches have been applied and co-created solutions are finalized for implementation. The selection criteria for control sites has been added to ensure comparability with intervention sites where the piloted solutions will be implemented. See pg. 10

**There is also need to start the criteria for the purposive sampling at the midline assessment.**

We have revised the sentences to reflect clearly the criteria for the purposive sampling at the midline assessment and other time points of the project. See pg. 11

**Study data quality control and assurance measures are wanting.**

This has been improved and revised on pg. 15

**Reviewer #3 comments:**

**The manuscript outlines a detailed and systematic protocol for assessing the effectiveness and implementation of the "Let’s Talk About Vaccines" project. Drawing from my extensive experience in global health security and epidemic intelligence, I find the use of CBPR and HCD methods both innovative and highly appropriate for addressing the critical issue of low childhood immunization rates in Mozambique and Malawi. The methodology is robust and well-documented, ensuring that the study can be replicated and will yield valuable insights.**

Thank you for your positive feedback.

**Here are a few minor recommendations for improvement:**

**Continuously review and update ethical considerations throughout the study, particularly concerning participant privacy and data sharing agreements, reflecting the dynamic nature of public health research.**

Thank you for this observation. We have added a relevant sentence to reflect this point in the ethics section. See pg. 18

**Provide more specific examples of successful applications of the HCD approach in similar contexts to better justify its use in this study. My work with various health security initiatives has shown the importance of such context-specific illustrations.**

Our justification for the use is that actually there is a dearth of rigorous research on HCD approaches for this type of programming, which is why we wanted to document and study the approach. We have added a few examples that applied HCD showing limited application in similar contexts. See pg. 4

**Address potential limitations related to the interrupted time series analysis, especially concerning the quality and completeness of routine immunization data. Drawing from my experience in managing large-scale health data projects, ensuring data integrity is crucial for accurate outcomes.**

We have added a detailed description of our our plan to assess and ensure data quality throughout our study and document any anomalies considering this perennial problem. See pg.15. We have also included a similar statement under limitations which also explains how we will overcome the stated limitations. See pg. 25

**Overall, the manuscript is comprehensive and well-prepared, setting a solid foundation for subsequent stages of the review process.**

We sincerely appreciate the positive feedback.

**Reviewer #4 comments: Background: Add more context:**

**1. Definition of vaccine coverage.** We have added the definition, see pg.2. For our project we measured this by full vaccination & dropout rate.

**2. Highlight the challenges of vaccine coverage.** We have added the challenges in the introduction and relevant references. See pg. 2

**3. Mention the challenges that the methods under study are aimed to address.** The project uses the various methods in trying to uncover the challenges to full immunization. See pg.3

**Methods: Study population:**

The study population has been revised in the methods section. See pg. 8 and 9

**Which** **vaccines are being studied? Are the challenges of vaccine coverage the same for all vaccines**?

All recommended vaccines administered to children under two are being studied. Challenges in vaccines vary depending on when they are administered and if multiple doses. The project being evaluated aims to unearth some of these challenges.

**Results/Dissemination:**

**Is health education/community dissemination in the plan?**

Dissemination of findings to various communities involved and stakeholders is in the plans. Health education may be suggested as part of the solutions co-designed in the HCD approach.

**Reviewer #5: Comments**

**Overall, relevant study area. The objectives can be explained more explicitly. The methodology, especially the sampling to be more detailed.**

We have refined our objectives to provide more explicit explanations. See pg. 6. We have also added more detailed information on our methodology, especially in the sampling section. See pages 10 to 11.

**Reviewer #6: comments**

**The author must explain rationale for the study more clearly, just referring to sub-optimal full immunization coverage and COVID-19 effect does not serve the purpose. It should define if there are some barriers affecting reaching to community, any particular beliefs etc. that hinder vaccination coverage.**

We have noted your suggestion and revised to include some related barriers. See introduction pg. 2

**Also what proposed intervention the Village Reach team expect, out of the HCD workshops planned? More clarity on possible intervention that may come out must be mentioned here. If this study has been conducted in other countries of LMIC/LIC, it would be useful to quote some examples.**

Some expected interventions have been added but this specific study hasn’t been conducted in other countries. See pg. 4

**Reviewer #7: Comments**

**Due to the process-oriented nature of the proposed analysis, the protocol as described in the current manuscript is not replicable. Given that replicability is the manuscript's primary objective, its contribution to the existing body of literature remains ambiguous.**

**A revision would be stronger if the methods were more specific and much more concise. The methodology may not lend itself to such a description. Remove didactic language. While many theories of evaluation are referenced it is unclear how they are all brought together into a cohesive analytic plan.**

We appreciate your insights and have made significant revisions to the protocol paper to enhance replicability and clarity of our methodology.

**It is unlikely that the study is powered appropriately to detect significance of impact on a dropout rate of 5–6%. A sample size calculation to see a difference of about 5% coverage usually needs around N=250. You would be looking for a change of 2%. The study would not need a 2-sided test unless there is a reason why the proposed interventions would increase dropout rates? Is the "adequate sample size" for the study as a whole, for each country or each site?**

Thank you for picking this up. We have revised the sample size calculation to indicate that it is a one-sided test as we do not anticipate an increase in dropout rates as a result of the intervention. See pg.11 and12. The analysis is done per country and thus the sample size of 300 is calculated for each country.

**The document does not describe the full timeline of the proposed study the timeline section states "until the end of 2024" when did the study start? How will the midpoint be determined?**

The timeline has been updated. See pg. 10

**Given that the currently only baseline and two assessment points, how can you stratify the model by month?**

Data is collected for every month although we have a baseline, midline and end point each of these time points has data spanning over a number of months. For example, for the baseline the data spans over a 12 month period.

**Refine the terminology around assumptions: in the introduction and in the assumptions section you are putting forward hypotheses. Assumptions are caveats and determinations that are not part of the formal study outcomes.**

Duly note and revised. See pg. 4

**Theory of change diagram: while rightly recognizing the complexity of healthcare utilization and delivery, the diagram and theory should reflect only the essential elements that are being tested or controlled in the study.**

Since we are using a systems thinking lens and acknowledge that the health system is a complex adaptive system we need to reflect on this to show factors at interplay when the adoption or scalability of such an approach is involved.

**While a good amount of the proposed data is theorized to come from the files of Village Reach, it is unclear whether all the proposed data will be available, and more information is needed to provide assurance that access to the data of interest will be available.**

As an embedded evaluation there is great scope for working with the project staff who will closely liaise with EPI but are also responsible for DHIS data collection within VillageReach. We have added some statement on this in the paper. See pg. 15

Yours sincerely

Corresponding author

Dr. Linda Shuro

Senior Researcher/ Post Doc Research Fellow

School of Public Health

University of Western Cape
